# Supplementary material for: How improvements to drug effectiveness impact mass drug administration for control and elimination of schistosomiasis
Source: PLoS Negl Trop Dis. 2025 Jun 2;19(6):e0012624. doi: 10.1371/journal.pntd.0012624 (PMC12129348; doi:10.1371/journal.pntd.0012624)
Supplement: Attachment — Submitted filename: renamed_d1681.pdf [file pntd.0012624.s001.pdf]

Reviewer's Comments

Title: How improvements to drug effectiveness impact mass drug administration for control and elimination of schistosomiasis (MS No.: PNTD-D-24-01468)

Overall: This study by Ellis and his colleagues is interesting and timely which is undertaken while deworming program using PZQ is in the progress. The authors investigated the impact of drug profile improvements to the current practice of using PZQ in MDA, employing an individual based stochastic mathematical model of the transmission and mass drug treatment of *S. mansoni* and *S. haematobium.* Finings of the study suggest that good coverage and improved drug efficacy best reduce prevalence and achieving interruption of transmission, but modest benefit of improved drug efficacy against juvenile parasite. The results from the mathematical models maybe be useful in designing new drug or improving formulation of the existing. Hence, the work is highly relevant and addresses topical issue.

The MS is also generally well written and structured. I recommend publication of the work in PLoS Negl Trop Dis journal after the authors have addressed the minor edits/comments indicated below.

Specific comments

Line 52: what does good MDA program mean? Coverage, frequency of distribution, drug quality?

Lines 67 &68: Replace in-text citation "World Health Organization, 2022" with No.7.

Line 68: italicize the genus name *Schistosoma*

Lines 343 & 344: In addition to WASH interventions as a key supplement to MDA, it is also important to consider snail control. This is important, particularly in endemic setting where animals are involved in transmission/maintenance of human schistosomes.

Lines 363 -366, Reference No. 6: check if it is in the in- text citation

Line 379, Reference # 10: provide correct citation for work by Wilson
