# Supplementary material for: How improvements to drug effectiveness impact mass drug administration for control and elimination of schistosomiasis
Source: PLoS Negl Trop Dis. 2025 Jun 2;19(6):e0012624. doi: 10.1371/journal.pntd.0012624 (PMC12129348; doi:10.1371/journal.pntd.0012624)
Supplement: Attachment — Submitted filename: Response to reviewers.docx [file pntd.0012624.s003.docx]

Dear Dr Liand, Dr Tamarozzi, Dr Kamhawi and Dr Brindley,

# Thank you for giving us the opportunity to submit a revised manuscript to PLOS NTDs. We thank the reviewers for their comments on our work. We have carefully considered each point raised and have made several revisions to improve the manuscript. We do not agree with all of the recommendations, as some of the perceived weaknesses of our study were already accounted for and have therefore instead made changes to clarify these key aspects. Below, we provide a detailed point-by-point response to each reviewer's comments in red text and explain the corresponding changes made to the manuscript. The line numbers correspond to the revised manuscript with tracked changes.

# Response to reviewers

## Reviewer #1:

This study by Ellis and his colleagues is interesting and timely which is undertaken while deworming program using PZQ is in the progress. The authors investigated the impact of drug profile improvements to the current practice of using PZQ in MDA, employing an individual based stochastic mathematical model of the transmission and mass drug treatment of S. mansoni and S. haematobium. Finings of the study suggest that good coverage and improved drug efficacy best reduce prevalence and achieving interruption of transmission, but modest benefit of improved drug efficacy against juvenile parasite. The results from the mathematical models maybe be useful in designing new drug or improving formulation of the existing. Hence, the work is highly relevant and addresses topical issue.

The MS is also generally well written and structured. I recommend publication of the work in PLoS Negl Trop Dis journal after the authors have addressed the minor edits/comments indicated below.

We thank the reviewer for their kind comments and taking the time to review the manuscript.

**Specific comments**

Line 52: what does good MDA program mean? Coverage, frequency of distribution, drug

quality?

We thank the reviewer for bringing this to our attention. We have clarified that in this context, we mean frequent treatment with high coverage in the target population (high percentage treated each round of MDA).

Lines 67 & 68: Replace in-text citation “World Health Organization, 2022” with No.7.

Thank you for spotting this error, it has been corrected.

Line 68: italicize the genus name Schistosoma

We have made this change.

Lines 343 & 344: In addition to WASH interventions as a key supplement to MDA, it is also

important to consider snail control. This is important, particularly in endemic setting where

animals are involved in transmission/maintenance of human schistosomes.

We thank the reviewer for making a good point and we have expanded the discussion to mention snail control alongside WASH (lines 352-353).

Lines 363 -366, Reference No. 6: check if it is in the in- text citation

We greatly appreciate the reviewer spotting this, this reference was no longer used but we had failed to remove it. We have now done so and updated the reference numbers.

Line 379, Reference # 10: provide correct citation for work by Wilson

This has now been corrected.

## Reviewer #2:

This manuscript explores the impact of improved drug efficacy on schistosomiasis mass drug administration (MDA) using a mathematical model. While the study has some novelty and public health relevance, the model suffers from several key methodological flaws that require significant revisions.

Major Weaknesses:

1.The model assumes perfect mixing within the population, neglecting heterogeneity in contact rates, activity patterns, and environmental exposures. This oversimplification likely biases the results and inflates the predicted effectiveness of interventions.

We appreciate the reviewer raising this important point about heterogeneity in infection. Our model does account for heterogeneity in population mixing through age-specific contact rates given in Tables 1 and 2. There is also variation between individuals, by predisposition to infection that is negative binomially distributed. This distribution is generated from a compounding of a Poisson distribution for each person where the mean of the Poisson across the population of people is a gamma distribution. We have added to the methods section (line 128) to make this more explicit.

2.The model uses idealized drug assumptions, particularly regarding the 24-week slow-release formulation, which lacks real-world evidence. Furthermore, it assumes similar drug mechanisms for juvenile and adult worms, disregarding potential differences in drug pharmacokinetics and mechanisms of action.

We thank the reviewer for this observation. The drug assumptions are indeed idealised; however, this is intentional as our study is designed to provide a guide to future drug development, rather than assess something that is currently in production. Given that the BMGF is considering supporting different pharmaceutical development for the treatment of schistosome parasites we explore what characteristics would be beneficial over those of the current treatment based on praziquantel. The model parameters can be changed to suit a specific drug when a likely candidate emerges. This is already explained in the manuscript, but we have expanded and made it clearer on this point in the introduction (lines 107-108) and discussion (lines 310-311).

We do not consider the specific mechanisms or pharmacokinetics for juveniles or adult worms, but we do not disregard the differences. We assumed that PZQ, the current drug used for SCH treatment, is completely ineffective against juveniles and then varied the efficacy of a potential new drug on the juveniles. The results we show in this paper compare no efficacy against juveniles with efficacy that is the same against juveniles as against adult worms. We have added to the methods (lines 151-153) to make this clearer.

3.Although the authors use two models, the validation focuses only on the consistency of main conclusions, not on thorough verification of the model’s parameterization, assumptions, and results across different scenarios. This limited validation is insufficient to address the model’s inherent limitations.

We thank the reviewer for highlighting this critical aspect. While this has been shown in previously published work (referenced on lines 115 and 132), we acknowledge that this context could have been better explained in the current manuscript. We have rewritten part of the methods section to cite the previous model validation that has been done and the scenarios in which these models have been used extensively previously (lines 132-141).

4.The model does not adequately account for real-world complexities of MDA implementation, such as poor drug adherence, population mobility, and the synergistic effects of multiple interventions. This disconnect limits the practical applicability of the findings.

These are very important factors in the transmission of SCH. Variation in drug adherence is covered in the model through the ‘never treated’ population. We acknowledge that there are more sophisticated ways to model this and have added this to the discussion (lines 170-173). For the purposes of this study, including a never treated population is sufficient to demonstrate how low adherence can negate the impact of a new drug and make the case that good to high coverage across the whole population (the degree dependent of the magnitude of R_0_) is necessary to reach elimination.

The aim of this study was to demonstrate the impact of different drug profiles for MDA in a single community. Population mobility is an important factor and work on this topic has recently been published by Collyer et al. (doi.org/10.1098/rstb.2022.0273) for STH. However, we believe that this is outside the scope of this study where we are considering a single community, although we mention in the discussion how constant reintroduction of infection through movement of people will affect the ability to eliminate SCH (lines 337-342).

Finally, as we are comparing MDA specifically, it is necessary to isolate this intervention. Other major interventions are the implementation of WASH and snail control. In both cases this would have the effect of reducing the infection rate in the model (lower the value of the effective reproductive number R), which would decrease the time taken to eliminate SCH. However, there is limited available data to be able to model these interventions effectively and therefore including them would add uncertainty to the model. We therefore made the decision to exclude these interventions and focus on isolating the difference with MDA alone, to show the impact of different drug profiles – which is the primary aim of this model based study. However, we have mentioned other interventions in the discussion which has been expanded to acknowledge the importance of snail control as well as WASH (lines 363-366).

Recommendation:

Given the aforementioned methodological shortcomings, I recommend a Major Revision. The authors must:

1.Thoroughly discuss the limitations of the model and clarify how these limitations may affect the results.
2.Revise or reconsider the drug assumptions to be more realistic, and perform sensitivity analysis.
3.Employ a more comprehensive validation approach to ensure the robustness of the model results.
4.Incorporate more real-world complexities in the model to improve its practical implications.

We thank the reviewer fir taking the time to review our manuscript and for raising important points. As many of these have been covered in the manuscript and in particular the referenced papers which address parameter estimation issues and various added complexity from acquired immunity, through predisposition to infection and spatial transmission structure, we feel that the study does not have the methodological shortcomings the reviewer describes. It is important to take into account the referenced work in the text which addresses many complexities in models and papers published previously by the authors. We have given a response and explanation for each of the comments above. However, we acknowledge that the manuscript could have better explained some of those points. Therefore, in response to the reviewer’s recommendations, we have made the following changes, which are also described in our response to each comment above:

1. Added an extra sentence to the introduction and the discussion to explain that the improvements to the drug profile are not meant to represent a drug currently in development (lines 107-108 and 310-311).
2. Clarified that predisposition to infection in the model leads to heterogeneity in contact rates (line 128) based on the compounding of Poisson distributions with a gamma distributed mean.
3. Expanding the methods section to better explain the models and the previously published work on them (lines 132-141).
4. Clarified how we treat the difference between the drug response of juvenile and adult worms (lines 151-153).
5. Expanded on how the model accounts for adherence and referenced alternative approaches to this issue that we and others have published (lines 170-173).
